# *TRPM8* genetic variant is associated with chronic migraine and allodynia

**DOI:** 10.1186/s10194-019-1064-2

**Published:** 2019-12-16

**Authors:** Yu-Hsiang Ling, Shih-Pin Chen, Cathy Shen-Jang Fann, Shuu-Jiun Wang, Yen-Feng Wang

**Affiliations:** 1 Department of Neurology, Neurological Institute, Taipei Veterans General Hospital, Taipei, Taiwan; 2 Faculty of Medicine, School of Medicine, National Yang-Ming University, Taipei, Taiwan; 3 Institute of Clinical Medicine, National Yang-Ming University, Taipei, Taiwan; 4 Brain Research Center, National Yang-Ming University, Taipei, Taiwan; 5 Department of Medical Research, Taipei Veterans General Hospital, Taipei, Taiwan; 6 Institute of Biomedical Science, Academia Sinica, Taipei, Taiwan

**Keywords:** Single nucleotide polymorphism, rs10166942, Pain sensitisation

## Abstract

**Background:**

Many single nucleotide polymorphisms (SNPs) have been reported to be associated with migraine susceptibility. However, evidences for their associations with migraine endophenotypes or subtypes are scarce. We aimed to investigate the associations of pre-identified migraine susceptibility loci in Taiwanese with migraine endophenotypes or subtypes, including chronic migraine and allodynia.

**Methods:**

The associations of six SNPs identified from our previous study, including *TRPM8* rs10166942, *LRP1* rs1172113, *DLG2* rs655484, *GFRA1* rs3781545, *UPP2* rs7565931, and *GPR39* rs10803531, and migraine endophenotypes, including chronic migraine and allodynia were tested. Significant associations in the discovery cohort were validated in the replication cohort. The adjusted odds ratios (aOR) were calculated after controlling for confounders.

**Results:**

In total, 1904 patients (mean age 37.5 ± 12.2 years old, female ratio: 77.7%) including 1077 in the discovery cohort and 827 in the replication cohort were recruited. Of them, 584 (30.7%) had chronic migraine. Of the 6 investigated SNPs, *TRPM8* rs10166942 T allele-carrying patients were more likely to have chronic migraine than non-T allele carriers in both discovery and replication cohorts and combined samples (33.7% vs. 25.8%, *p* = 0.004, aOR = 1.62). In addition, T allele carriers reported more allodynic symptoms than non-T allele carriers (3.5 ± 3.7 vs. 2.6 ± 2.8, *p* < 0.001). However, allodynia severity did not differ between episodic and chronic migraine patients. No further correlations between genetic variants and endophenotypes were noted for the other SNPs.

**Conclusions:**

*TRPM8* may contribute to the pathogenesis of chronic migraine. However, our study did not support allodynia as a link between them. The underlying mechanisms deserve further investigations.

## Introduction

Migraine, which is characterised by recurrent pulsatile headaches associated with nausea, vomiting, photophobia, and phonophobia, is a common yet disabling disease [1] that can be clinically diagnosed using the proposed criteria in the International Classification of Headache Disorders (ICHD) [2]. Migraine is considered as a complex genetic disorder. Studies of twins and familial aggregation analyses indicate a strong genetic component in migraine, showing a heritability of 0.34–0.81 [3–8] that can be attributed to polygenes with a modest effect [9, 10]. Several single nucleotide polymorphisms (SNPs) associated with migraine susceptibility were recently identified by genome-wide association studies (GWAS) [11]. In our study among the Han Chinese population in Taiwan, several novel variants were identified to be associated with migraine in a two-stage GWAS [12], including rs655484 in *disks large homolog 2 (DLG2)* and rs3781545 in *GDNF family receptor alpha-1 (GFRA1*), rs10803531 in *G protein-coupled receptor 39* (*GPR39*), and rs7565931 in *uridine phosphorylase 2* (*UPP2*). Furthermore, the association between migraine and rs10166942 in *transient receptor potential melastatin 8* (*TRPM8*) as well as rs1172113 in *low density lipoprotein receptor-related protein 1 (LRP1*), the two most replicated SNPs in Caucasians, were also reproduced in our study cohort. The risk allele frequencies are listed in Additional file 1: Table S1.

Endophenotypes are clinical symptoms that differentiate patients into subdivisions with underlying genetic pathogenesis. Several endophenotypes have been proposed in migraine, including prodromal symptoms such as yawning, aura, or accompanying symptoms like nausea, vomiting and pulsating. Unilateral autonomic symptoms during attacks are also raised as endophenotypes of migraine [13]. The association of some endophenotypes and migraine implicit genotypes have been studied and reported [14, 15]. Among all migraine endophenotypes or subtypes, chronic migraine is the one with critical clinical significance because it locates at the end of more severe disease-related disability with a poorer quality of life [16]. The prevalence of chronic migraine ranges from 1.0% to 1.7% in Asian populations [17, 18]. However, no susceptible gene has been identified to be associated with chronic migraine to date. On the other hand, allodynia is another widely-studied endophenotype of migraine. Defined as the pain triggered by a normally innocuous stimulation, cutaneous allodynia is very common in migraine patients [19–21], especially in patients with chronic migraine [22]. The presence of allodynia often represents the peripheral, central, and disinhibitory sensitisation of pain pathways in affected patients [23].

In the present study, we aimed to investigate the association between migraine endophenotypes, especially for chronic migraine and allodynia, and known susceptible genes of migraine in Taiwan. Other investigated endophenotypes included aura and migrainous features. The candidate genes were chosen based on the findings of our previous migraine GWAS, the only published study in Asians [12]. The current study adopted a two-stage design, including discovery and replication cohorts of patients with migraine. In the replication cohort, we also evaluated the cutaneous allodynia profile in patients with migraine using a 17-item questionnaire, aiming to obtain the evidence for the association between genetic variants and cutaneous allodynia, a clinical marker signaling the sensitisation of trigeminovascular system and a potential predictor of migraine chronification [19].

## Materials and methods

### Ethics

The study was approved by the Institutional Review Board of the Taipei Veterans General Hospital, Taipei, Taiwan (IRB No. 2011–11-002GA). Written informed consent was obtained from each participant prior to entering the trial. All clinical investigations were conducted according to the principles expressed in the Declaration of Helsinki. All collected information was de-identified before statistical analysis. The corresponding author had full access to all of the data in the study and had final responsibility for the decision to submit the study for publication.

### Study participants and data collection

This was a two-stage study consisting of a discovery cohort and a replication cohort. All patients with migraine were recruited from the headache clinic of Taipei Veterans General Hospital, Taipei, Taiwan. The discovery and replication cohorts were recruited based on the time they entered the study and whether they completed the allodynia assessment. For the discovery and replication cohorts, we prospectively recruited patients from January 2011 to December 2014 and January 2014 to May 2017 respectively. The sample size was reached based on the numbers of unrelated participants during the study period. All participants completed a detailed headache intake form prior to joining the study and then partook in a semi-structured interview conducted by headache specialists; the participants’ demographics, headache characteristics and endophenotypes, medical history, and mental condition assessment were obtained during the interview. The diagnoses of migraine and chronic migraine were made based on the ICHD-3 criteria [2]. The level of anxiety and depression was measured by the Hospital Anxiety and Depression Scale (HADS); the quality of sleep was measured by the Pittsburgh Sleep Quality Index (PSQI).

### Genotyping

Based on the finding of our prior work [12], all participants in discovery cohort were genotyped for six SNPs known to be associated with migraine in Taiwanese, including rs10166942 in *TRPM8*, rs1172113 in *LRP1*, rs7565931 in *UPP2*, rs10803531 in *GPR39*, rs655484 in *DLG2*, and rs3781545 in *GFRA1*. In the replication cohort, every participant was genotyped for rs10166942 in *TRPM8—*the only significant one identified in the discovery cohort*.* The genotyping was conducted using the Sequenom MassARRAY iPLEX platform (Sequenom Inc., San Diego, CA, USA) in collaboration with the National Centre for Genomics Medicine, Academia Sinica, Taiwan. Of note, not all the genetic variants identified from migraine GWAS in Western populations were genotyped in this study because our previous study has demonstrated that except for the SNPs in *TRPM8* and *LRP1*, none of the other variants could be replicated in our population with this sample size [12].

### Allodynia assessment

We assessed the allodynia profile of all participants in the replication cohort. Using the 17-item allodynia assessment questionnaire specific for migraine patients, the participants were asked to recall any allodynic symptoms they had during a migraine attack [24]. The items were as followed: (1) combing hair; (2) pulling hair backward (example: ponytail); (3) shaving face; (4) wearing glasses; (5) wearing contact lens; (6) wearing earrings; (7) wearing necklaces; (8) wearing anything on head or neck; (9) wearing anything on arms or wrists; (10) wearing a finger ring; (11) wearing tight clothes; (12) wearing a watch; (13) being covered by a heavy blanket; (14) pouring water onto the face; (15) resting the face ipsilateral to the headache side during a headache attack; (16) being exposed to heat (e.g., cooking); and (17) being exposed to cold (e.g., breathing through the nose on a cold day). The total allodynia score was calculated to represent the severity of allodynia by summing up all allodynia symptoms for one point each.

### Statistical analysis

The descriptive statistics were presented as the mean ± standard deviation (SD), or as percentages. Unanswered queries in the questionnaire were assigned as missing values and excluded from subsequent analyses. Categorical variables were compared between groups using the chi-square test. Continuous variables were compared between groups using *Student t*-test or one-way analysis of variance (ANOVA). Based on the genotyping results, the participants were classified into two groups: (1) risk allele carriers, and (2) non-risk allele carriers. The odds ratios were calculated for risk allele carriers versus the non-carriers. The associations between genotypes and phenotypes were analyzed; significant associations were further calculated using a general linear model and a logistic regression model. The validation of regression model was assessed using mean square error (MSE) to evaluate the fitness of our model. All statistical analyses were performed on SPSS version 23 (IBM, Armonk, NY, USA) and SAS version 9.4 (SAS Inc., Cary, NC, USA). Statistical significance was defined as *p* < 0.05, or as adjusted for the multi-comparison Bonferroni correction.

### Data availability

The data that support the findings of this study are available from the corresponding author on reasonable request.

## Results

### Participants

**Fig. 1:**
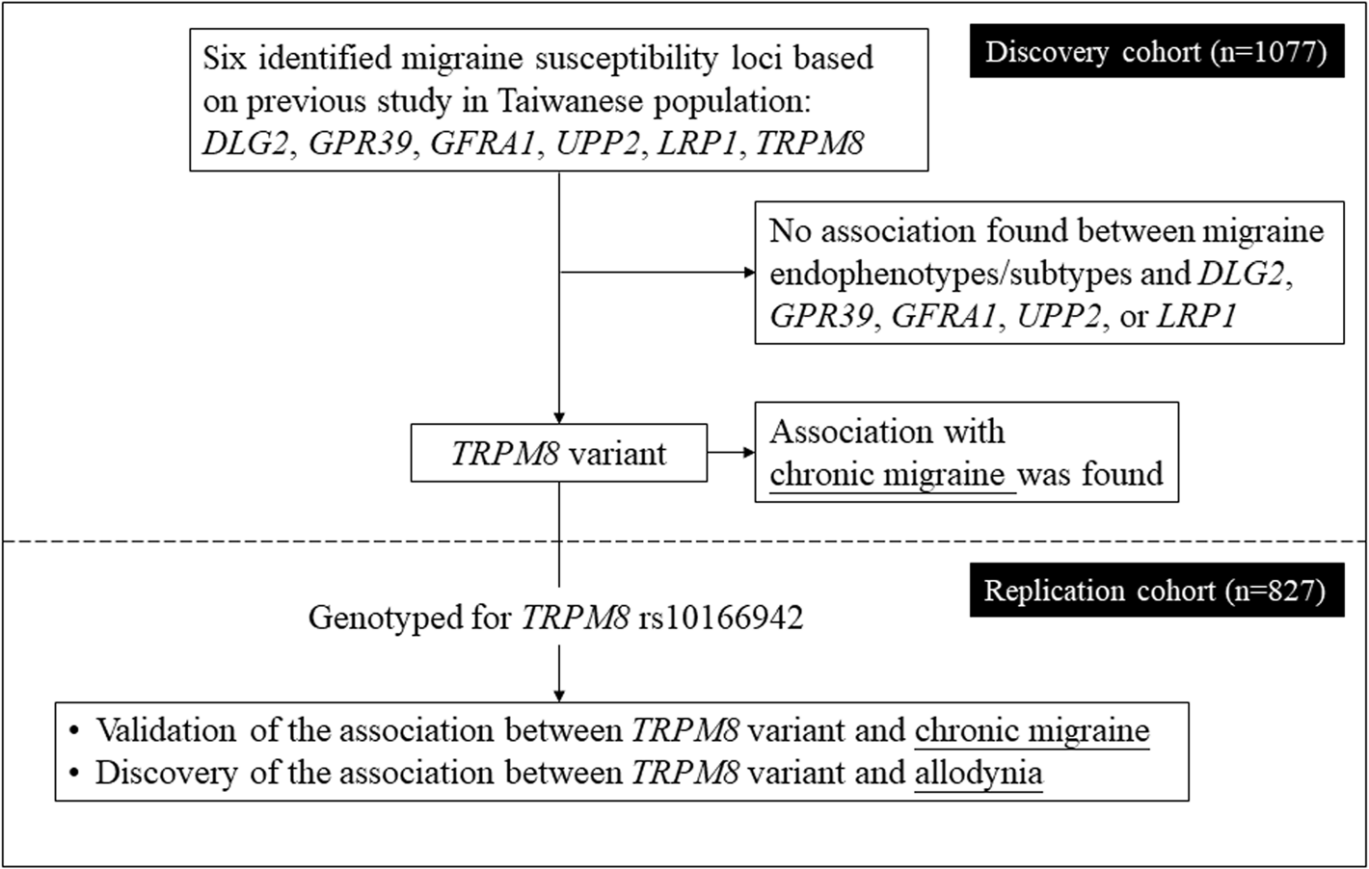
Study design and main findings of the study

In this two-stage genetic association study, we recruited 1077 and 827 patients with migraine in the discovery and replication cohorts, respectively to investigate the associations between chronic migraine and candidate genes (Fig. 1). Overall, 340 (17.9%) had aura, and 584 (30.7%) were diagnosed with chronic migraine. The demographics are shown in Table 1.

**Table 1:** Baseline demographics and clinical data of participants

	Discovery cohort (*n* = 1077)	Replication cohort (*n* = 827)	*p* value	Combined (*n* = 1904)
Age	37.9 ± 12.6	37.0 ± 11.7	0.116	37.5 ± 12.2
Female sex	827 (76.8)	653 (79.0)	0.259	1480 (77.7)
Presence of aura	180 (16.7)	160 (19.3)	0.137	340 (17.9)
Chronic migraine	332 (30.8)	252 (30.5)	0.868	584 (30.7)
Disease duration of migraine (years)	18.4 ± 11.2	18.1 ± 11.1	0.591	18.2 ± 11.1
HADS- anxiety score	7.6 ± 4.0	7.4 ± 4.1	0.176	7.5 ± 4.0
HADS- depression score	5.4 ± 3.7	5.1 ± 3.7	0.087	5.2 ± 3.7
PSQI- sleep score	8.8 ± 4.0	9.0 ± 4.0	0.235	8.9 ± 4.0

Data were presented as *n* (%) or mean ± SD; N: number of participants; HADS: hospital anxiety and depression scale; PSQI: Pittsburgh sleep quality index

### Correlation of genotypes and endophenotypes of migraine

We genotyped all six susceptible variants in all participants and explored their possible associations with endophenotypes of migraine. Among the six investigated SNPs, we found that only *TRPM8* variant rs10166942 was associated with chronic migraine. Therefore, this positive association was further investigated by examining all potential confounders. We did not find the association between the rest of genotypes and endophenotypes, including aura, chronic migraine as well as migrainous features (results shown in Additional file 1: Table S2).

### Correlations of rs10166942 with chronic migraine

The proportions of chronic migraine in *TRPM8* rs10166942 T allele-carrying patients were significantly higher than non-carriers in either discovery or replication cohorts as well as combined results (33.7% vs. 25.8%, *p* < 0.001), and the results remained significant after Bonferroni corrections (Table 2).

**Table 2:** Migraine endophenotypes and *TRPM8* genotyping in participants

	Discovery cohort (*N* = 1077)			Replication cohort (*N* = 827)			Combined
	T/T or T/C *n* = 681 (63.2%)	C/C *n* = 396 (36.8%)	*p* value	T/T or T/C *n* = 495 (59.8%)	C/C *n* = 332 (40.2%)	*p* value	*p* value
Age	38.2 ± 12.8	37.3 ± 12.2	0.219	37.1 ± 11.9	36.9 ± 11.4	0.861	0.267
Gender- female	515 (75.6)	312 (78.8)	0.236	398 (80.4)	255 (76.8)	0.214	0.899
Presence of aura	102 (15.0)	78 (19.7)	0.045	91 (18.4)	69 (20.8)	0.392	0.036
Chronic migraine	228 (33.5)	104 (26.3)	0.013	168 (33.9)	84 (25.3)	0.008	< 0.001^*^
Unilaterality	513 (75.3)	303 (76.5)	0.662	406 (82.0)	273 (82.2)	0.939	0.676
Pulsatility	500 (74.5)	291 (74.4)	0.974	386 (78.0)	256 (77.1)	0.768	0.923
Aggravation by or avoidance of physical activity	599 (89.4)	346 (89.2)	0.908	441 (89.1)	276 (83.1)	0.013	0.057
Nausea	589 (87.5)	350 (89.3)	0.389	445 (89.9)	297 (89.5)	0.838	0.823
Vomiting	305 (45.3)	177 (45.2)	0.958	263 (53.1)	165 (49.7)	0.333	0.826
Photophobia	342 (50.8)	188 (48.1)	0.390	246 (49.7)	158 (47.6)	0.552	0.512
Phonophobia	507 (75.3)	294 (75.0)	0.903	359 (72.5)	234 (70.5)	0.523	0.839
Allodynia severity	–	–	–	3.5 ± 3.7	2.6 ± 2.8	< 0.001^*^	–

Data were presented as *n* (%) or mean ± SD. T/T: TT homozygous group; T/C: TC or CT heterozygous group; C/C: CC homozygous group; HADS: hospital anxiety and depression scale; * significant after Bonferroni correction (*p* < 0.05/12 = 0.0042); allodynia severity was presented with the total allodynia scores

The *TRPM8* rs10166942 T allele was still independently associated with chronic migraine (adjusted odds ratio = 1.62, *p* = 0.004) after controlling for age, gender, education, body mass index, depression and anxiety using a multivariable logistic regression (Table 3) in both discovery and replication cohorts.

**Table 3:** Demonstration of the associations between chronic migraine and rs10166942 genotypes by logistic regression models

	Univariate		Multivariable^a^	
	OR	*p* value	OR	*p* value
*Discovery cohort*				
Age	1.03	< 0.001^**^	1.02	0.001^**^
Sex (female = 1/male = 0)	1.29	0.116	–	–
Education (years)	0.92	< 0.001^**^	0.83	< 0.001^**^
Level of anxiety	1.06	< 0.001^**^	–	–
Level of depression	1.09	< 0.001^**^	–	–
rs10166942 (T carriers =1)	1.41	0.014^*^	1.40	0.020^*^
*Replication cohort*				
Age	1.04	< 0.001^**^	1.02	0.006^**^
Sex (female = 1/male = 0)	1.28	0.194	–	–
Education (years)	0.84	< 0.001^**^	0.70	< 0.001^**^
BMI	1.01	0.517	–	–
Level of anxiety	1.05	0.008^**^	–	–
Level of depression	1.11	< 0.001^**^	1.10	0.001^**^
rs10166942 (T carriers =1)	1.52	0.008^**^	1.56	0.008^**^
*Combined*				
Age	1.03	< 0.001^**^	1.02	0.019^*^
Sex (female = 1/male = 0)	1.29	0.042^*^	–	–
Education (years)	0.89	< 0.001^**^	0.71	< 0.001^**^
BMI	1.01	0.529	–	–
Level of anxiety	1.06	< 0.001^**^	–	–
Level of depression	1.09	< 0.001^**^	1.11	< 0.001^**^
rs10166942 (T carriers =1)	1.46	0.001^**^	1.62	0.004^**^

^a^controlled by age, sex and anxious level. *OR* odds ratio; *BMI* body mass index; level of anxiety: presented by HADS anxiety score; level of depression: presented by HADS depression score. ^*^ indicated *p* < 0.05; ^**^ indicated *p* < 0.01. Of note, BMI data were not available in discovery cohort

### Allodynia assessment

Primarily, the total allodynia score was higher in the migraine participants carrying the T allele than in those without (3.5 ± 3.7 vs. 2.6 ± 2.8, *p* < 0.001) (Table 2). After controlling for age, gender, chronic migraine, disease duration of migraine, and level of anxiety and depression (HADS anxiety and depression score), rs10166942 T allele carriers were still associated with total allodynia scores versus non-T allele carriers (*p* = 0.001, Table 4). Of note, we did not find significant difference in the severity of allodynia between participants with and without chronic migraine (3.2 ± 3.2 vs. 3.1 ± 3.4, *p* = 0.623). In addition, we found that T allele carriers were more susceptible to allodynia that was provoked by wearing eyeglasses, wearing contact lenses, wearing earrings, wearing necklaces, wearing anything on the wrist or forearm, wearing a ring, pouring water on the face, exposure to heat, and breathing through nose on cold days than homozygous C allele carriers (Additional file 1: Table S3).

**Table 4:** Demonstration of the associations between allodynia severity and rs10166942 genotypes by general linear models

	Univariate		Multivariable^a^	
	B	*p* value	B	*p* value
Age	0.008	0.443	−0.035	0.019^*^
Sex (female = 1/male = 0)	1.230	< 0.001^**^	1.079	< 0.001^**^
Chronic migraine	0.126	0.623	–	–
Disease duration of migraine (years)	0.032	0.003^**^	0.054	0.001^**^
Level of anxiety	0.135	< 0.001^**^	0.103	0.007^*^
Level of depression	0.108	0.001^**^	–	–
rs10166942 (T carriers =1)	0.888	0.001^**^	0.776	0.001^**^

^a^controlled by age, sex and depressive level. *B* B coefficient; level of anxiety was presented by HADS anxiety score; level of depression was presented by HADS depression score. ^*^p < 0.05; ^**^p < 0.01

**Fig. 2:**
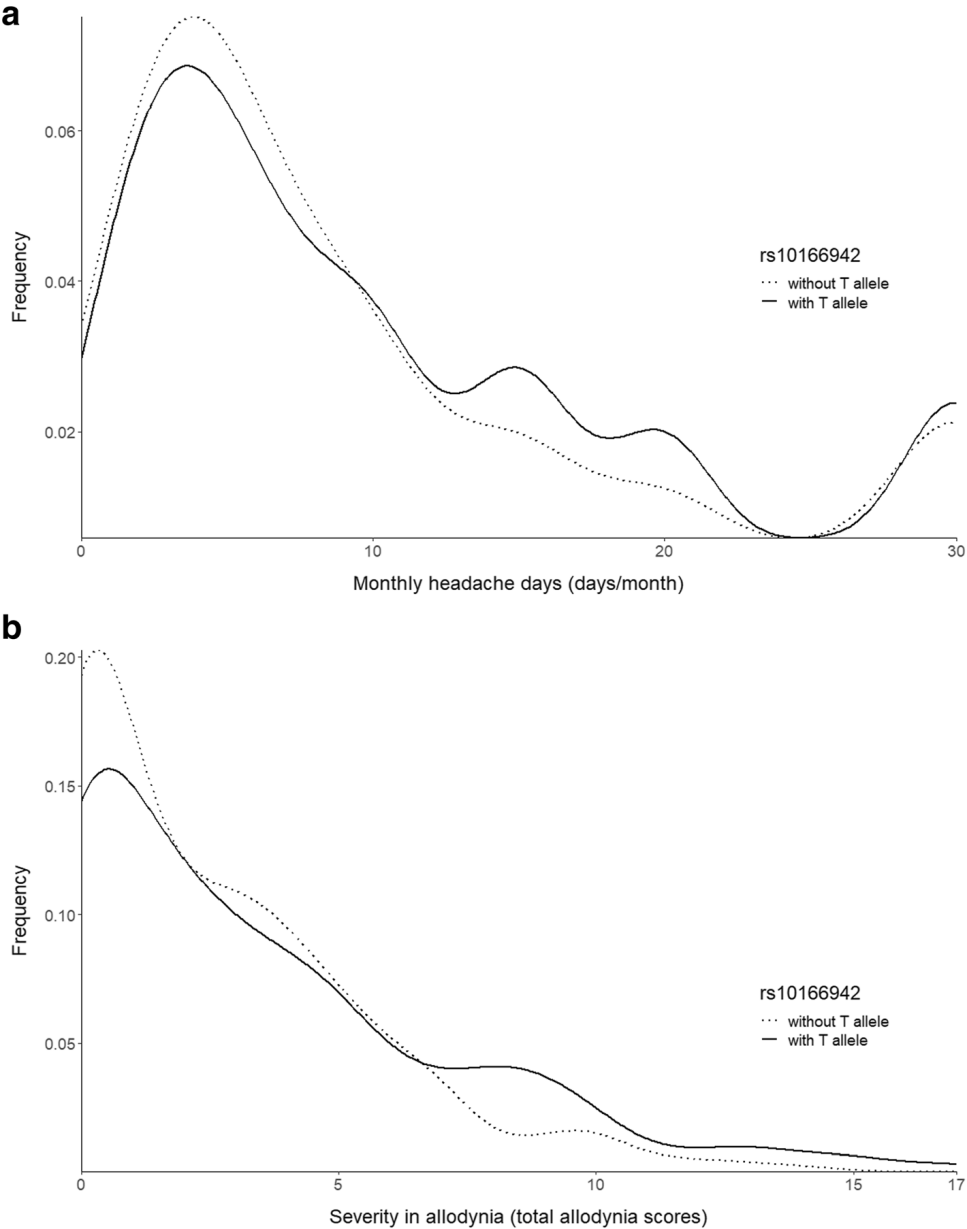
**a** The distribution of headache frequencies in migraineurs with or without T allele in rs10166942. **b** The distribution of allodynia severity in migraineurs with or without T allele in rs10166942

The distribution of headache frequencies and allodynia severity in migraineurs with or without T allele rs10166942 is illustrated in Fig. 2, showing that patients with T allele in rs10166942 tend to have a higher headache frequency or more severe allodynia than those without.

## Discussion

To the best of our knowledge, this study first demonstrates that the *TRPM8* rs10166942 T allele–also known as the risk allele of migraine–makes migraine patients more susceptible to migraine chronification. This finding was confirmed by a two-stage validation with two independent cohorts. In addition, our study also demonstrates the association between the *TRPM8* rs10166942 T allele and allodynia severity. However, our study did not find a difference in allodynia severity between patients with episodic and chronic migraine.

The pathophysiology of chronic migraine is an urgent issue to be solved. Prior attempts to search for the relationship between genetic polymorphism, including another *TRPM8* variant rs17862920, and chronic migraine have failed [25]. However, our study demonstrated an association between the rs10166942 T allele and chronic migraine with an adjusted odds ratio of 1.62, even after controlling for other known confounding factors. This finding deserves verification by following up with patients with episodic migraine in a longitudinal design. Several factors are considered as being involved in the process of migraine chronification, including older age, female gender, lower educational status, obesity, depression, stress and poor response to acute treatment [26]. In line with prior studies, our data showed that older age, lower education and higher depressive levels were risk factors for chronic migraine.

In animal models of craniofacial pain, two studies have demonstrated the association between TRPM8 and allodynia [27, 28]. However, the association between the *TRPM8* genetic variant and allodynia has never been reported in human studies with migraine. Our study can be considered as a translational research, i.e. from animal models to human patients. Our data showed that severity of allodynia in migraineurs was positively associated with being female, longer disease duration of migraine, and psychiatric comorbidities, including depression and anxiety. These findings were also consistent with previous studies [29, 30], indicating that our cohort is a representative sample for migraine.

Of note, unlike a previous study [30], our data did not show associations between chronic migraine and the severity of allodynia. In fact, the results of the associations between allodynia and chronic migraine remained undetermined. One paediatric study also did not demonstrate an association between allodynia and chronic migraine [31]. In addition, one study showed that the threshold of pressure pain as measured by quantitative sensory tests did not differ between women with episodic and chronic migraine [32]. Recent results from MAST (Migraine in America Symptoms and Treatment) study showed that increased headache frequency was associated with allodynia; however, the effect was attenuated after controlling for sociodemographic profiles [33]. Regardless of the discrepancy, our results showed that allodynia was positively associated with the disease duration of migraine, which is in line with a previous study [21].

Encoded by gene *TRPM8*, the TRPM8 receptor is a non-selective cation channel that serves as the primary sensor of cold and cold-induced pain in mammals. The receptor can be activated by cold ranging from 8 to 28 °C and cooling agents, including methanol and icilin [34]. The T allele in the *TRPM8* variant rs10166942 is a risk allele for migraine, and our results further indicate that it is also a risk allele to allodynia in migraineurs. The association between the *TRPM8* variant rs10166942 and migraine was initially found in Western populations and replicated later in Asians [12]; here, the T allele was the risk allele for migraine across all studies [11]. To date, evidence in level of molecular mechanism that determines the functional effect of rs10166942 is lacking. Based on computational predictions [35], rs10166942 is located at the regulatory region of *TRPM8*, the functional effect of it might be alterations in the transcriptional regulation of TRPM8 and thus the affecting the phenotypes of patients. Another possibility is that rs10166942 has a strong linkage disequilibrium with the true causal variant, which remains to be identified. A recent study [36] demonstrated that the frequency of T allele in rs10166942 is positively corelated to the latitude and climate changes, suggesting that T allele-carrying genetic variant *TRPM8* played a role in adaption to cold temperatures.

We speculate that T allele of rs10166942 increases the expression level of TRPM8, sensitizes humans’ cold sensation, helping them survive from the extreme changes in temperature in high latitude regions. On the other hand, T allele in rs10166942 may be associated with the functional changes of TRPM8 that contribute to the hypersensitivity to cold sensation and temperature changes. Pathways that transmit signals of cold sensation, including trigeminothalamic tracts, are repeatedly stimulated by cold and temperature changes, and eventually become sensitized in such individuals. The central sensitization of trigeminothalamic system may lead to worsening of cutaneous allodynia, especially to temperature-related stimulations like our investigated cohort reported, and to migraine progression and chronification. Of course, to explore the hypothesis, the exact impact of genotype rs10166942 on TRPM8 expression needs to be elucidated.

The association between *TRPM8* and allodynia has been investigated in animal models, but the results are controversial. Kayama et al. revealed that the activation of TRPM8 reversed the heat allodynia in a mouse meningeal inflammation model, indicating that the activation of TRPM8 is protective against allodynia in migraine [28]. The same study also introduced a cell culture model with coexpression of TRPM8 and transient receptor potential cation channel subfamily V member 1 (TRPV1), showing that the activation of TRPV1, which would lead to heat and mechanical allodynia [37], was attenuated by the activation of TRPM8. The work of Kayama et al. suggests that the pathogenesis of allodynia involves multiple nociceptors, which could partially explain how the genetic variant of the cold receptor TRPM8 could be associated with both thermal and mechanical allodynia. Another study showed that the activation of meningeal TRPM8 receptors by the TRPM8 agonist icilin was associated with facial and hind paw mechanical allodynia in rats [27]. The discrepancy in results might be derived from different model systems (topical ilicin in rats vs. inflammatory soup in mice) with different readouts (mechanical vs. thermal allodynia).

Our study has limitations. First, this was a cross-sectional study. Therefore, the causal relationship of migraine chronification and *TRPM8* variant could not be ascertained. Based on our findings, a longitudinal study is warranted to elucidate the association between the *TRPM8* genetic variant rs10166942 and the evolution of migraine. Second, recall bias of allodynia could have been introduced because not all participants were interviewed during headache attacks. Third, the current study was a single hospital-based study despite validation with another independent cohort; therefore, the results might not be generalisable to other migraine populations. Last, the sample size of the present study was limited, and only 6 migraine susceptible SNPs were tested considering a priori evidences (as indicated in the *Methods*) and the limitation of resources. However, the 6 tested SNPs were proven to be significant in the studied population, which makes our finding indicative, especially in Asian population. Indeed, further investigations of the association between *TRPM8* variant and chronic migraine in different populations are warranted.

## Conclusions

Our study shows that the *TRPM8* variant rs10166942 is associated with chronic migraine and allodynia in patients with migraine. Further investigation regarding the role of *TRPM8* in allodynia pathogenesis and migraine chronification may provide a novel treatment strategy.

## Supplementary information

**Additional file 1: Table S1.** The risk allele frequencies of investigated SNPs. **Table S2a**. Migraine endophenotypes and *LRP1* rs1172113 genotyping **Table S2b**. Migraine endophenotypes and *DLG2* rs655484 genotyping **Table S2c**. Migraine endophenotypes and *GFRA1* rs3781545 genotyping **Table S2d**. Migraine endophenotypes and *UPP2* rs7565931 genotyping. **Table S2e**. Migraine endophenotypes and *GPR39* rs10803531 genotyping. **Table S3**. Allodynia symptoms during migraine attacks

## Data Availability

The data that support the findings of this study are available from the corresponding author on reasonable request.

## Abbreviations

ANOVA: Analysis of variance
aOR: Adjusted odds ratio
DLG2: Disks large homolog 2
GFRA1: GDNF family receptor alpha-1
GPR39: G protein-coupled receptor 39
GWAS: Genome-wide association study
HADS: Hospital Anxiety and Depression Scale
ICHD: International classification of headache disorder
LRP1: Low density lipoprotein receptor-related protein 1
MAST: Migraine in America Symptoms and Treatment
MSE: Mean square error
PSQI: Pittsburgh sleep quality index
SD: Standard deviation
SNP: Single nucleotide polymorphism
TRPM8: Transient receptor potential melastatin 8
TRPV1: Transient receptor potential cation channel subfamily V member 1
UPP2: Uridine phosphorylase 2
