# Supplementary material for: TRPM8 genetic variant is associated with chronic migraine and allodynia
Source: J Headache Pain. 2019 Dec 16;20(1):115. doi: 10.1186/s10194-019-1064-2 (PMC6916225; doi:10.1186/s10194-019-1064-2)
Supplement: Supplementary file 1 — Additional file 1: Table S1. The risk allele frequencies of investigated SNPs. Table S2a. Migraine endophenotypes and LRP1 rs1172113 genotyping Table S2b. Migraine endophenotypes and DLG2 rs655484 genotyping Table S2c. Migraine endophenotypes and GFRA1 rs3781545 genotyping Table S2d. Migraine endophenotypes and UPP2 rs7565931 genotyping. Table S2e. Migraine endophenotypes and GPR39 rs10803531 genotyping. Table S3. Allodynia symptoms during migraine attacks [file 10194_2019_1064_MOESM1_ESM.docx]

**Table 1. The risk allele frequencies of investigated SNPs.**

| SNPs |  | Risk Allele |  | Risk Allele Frequency | | |
| --- | --- | --- | --- | --- | --- | --- |
|  |  |  |  | Taiwanese Migraineurs^1^ |  | Global^2^ |
| *TRPM8* rs10166942 |  | T |  | 0.39 |  | 0.63 |
| *LRP1* rs1172113 |  | T |  | 0.78 |  | 0.66 |
| *DLG2* rs655484 |  | C |  | 0.95 |  | 0.99 |
| *GFRA1* rs3781545 |  | G |  | 0.87 |  | 0.87 |
| *GPR39* rs10803531 |  | C |  | 0.97 |  | 0.20 |
| *UPP2* rs7565931 |  | C |  | 0.23 |  | 0.19 |

^1^ Chen et al., Genome-wide association study identifies novel susceptibility loci for migraine in Han Chinese resided in Taiwan. *Cephalalgia*, 2018; 38(**3**), 466–475.

^2^ Adapted from gnomAD database v2.1.1

**Table 2a. Migraine endophenotypes and *LRP1* rs1172113 genotyping**

|  | **C/C**  n=51 | **C/T or T/C**  n=368 | **T/T**  n=658 |  | *p value* |
| --- | --- | --- | --- | --- | --- |
| Age | 37.4 ± 11.3 | 37.4 ± 12.9 | 38.2 ± 12.5 |  | 0.618 |
| Female sex | 43 (84.3) | 289 (78.5) | 495 (75.2) |  | 0.207 |
| Presence of aura | 7 (13.7) | 64 (17.4) | 109 (16.6) |  | 0.795 |
| Chronic migraine | 21 (41.2) | 103 (28.0) | 208 (31.6) |  | 0.126 |
| Unilaterality | 32 (62.7) | 283 (76.9) | 501 (76.1) |  | 0.081 |
| Pulsatility | 37 (72.5) | 267 (73.8) | 487 (75.0) |  | 0.858 |
| Aggravation by or avoidance of physical activity | 46 (93.9) | 317 (87.8) | 582 (89.8) |  | 0.351 |
| Nausea | 46 (90.2) | 316 (87.1) | 577 (88.6) |  | 0.681 |
| Vomiting | 21 (41.2) | 156 (43.0) | 305 (46.9) |  | 0.412 |
| Photophobia | 24 (47.1) | 174 (47.9) | 332 (51.1) |  | 0.582 |
| Phonophobia | 40 (78.4) | 273 (75.2) | 488 (75.0) |  | 0.858 |

Data were presented as n (%) or mean ± SD. T/T: TT homozygous group; T/C: TC or CT heterozygous group; C/C: CC homozygous group; significant level set at 0.05/11=0.005 after Bonferroni corrections for multiple comparisons.

**Table 2b. Migraine endophenotypes and *DLG2* rs655484 genotyping**

|  | Discovery cohort (N=1077) | | |  | Replication cohort (N=824) | | |  | Combined |
| --- | --- | --- | --- | --- | --- | --- | --- | --- | --- |
|  | C/T or T/C  n= 50 (4.6%) | C/C  n= 1027 (95.4%) | *p* value |  | C/T or T/C  n=146 (17.7%) | C/C  n=678 (82.3%) | *p* value |  | *p* value |
| Age | 43.5 ± 14.9 | 37.6 ± 12.4 | 0.001 |  | 38.4 ± 12.5 | 36.7 ± 11.4 | 0.120 |  | 0.008 |
| Female sex | 39 (78.0) | 788 (76.7) | 0.835 |  | 119 (81.5) | 531 (78.3) | 0.392 |  | 0.300 |
| Presence of aura | 7 (14.0) | 173 (16.8) | 0.598 |  | 24 (16.4) | 136 (20.1) | 0.316 |  | 0.425 |
| Chronic migraine | 23 (46.0) | 309 (30.1) | 0.017 |  | 44 (30.1) | 207 (30.5) | 0.925 |  | 0.260 |
| Unilaterality | 39 (78.0) | 777 (75.7) | 0.706 |  | 124 (84.9) | 554 (81.7) | 0.355 |  | 0.099 |
| Pulsatility | 38 (76.0) | 753 (74.4) | 0.801 |  | 120 (82.2) | 520 (76.7) | 0.148 |  | 0.102 |
| Aggravation by or avoidance of physical activity | 42 (84.0) | 903 (89.6) | 0.212 |  | 127 (87.0) | 588 (86.7) | 0.933 |  | 0.364 |
| Nausea | 44 (88.0) | 895 (88.2) | 0.970 |  | 131 (89.7) | 608 (89.7) | 0.985 |  | 0.831 |
| Vomiting | 18 (36.0) | 464 (45.7) | 0.178 |  | 74 (50.7) | 352 (51.9) | 0.787 |  | 0.738 |
| Photophobia | 24 (48.0) | 506 (49.9) | 0.793 |  | 71 (48.6) | 332 (49.0) | 0.941 |  | 0.779 |
| Phonophobia | 32 (64.0) | 769 (75.8) | 0.060 |  | 104 (71.2) | 486 (71.7) | 0.913 |  | 0.154 |

Data were presented as n (%) or mean ± SD. T/C: TC or CT heterozygous group; C/C: CC homozygous group; significant level set at 0.05/11=0.005 after Bonferroni corrections for multiple comparisons. Note: no T/T homozygous participant was identified.

**Table 2c. Migraine endophenotypes and *GFRA1* rs3781545 genotyping**

|  |  | Discovery cohort (N=1077) | | |  |  | Replication cohort (N=667) | | |  | Combined |
| --- | --- | --- | --- | --- | --- | --- | --- | --- | --- | --- | --- |
|  | AA  n= 33  (3.1%) | G/A or A/G  n= 289  (26.8%) | G/G  n= 755  (70.1%) | *p* value |  | AA  n= 33  (4.9%) | G/A or A/G  n= 111  (16.6%) | G/G  n= 523  (78.4%) | *p* value |  | *p* value |
| Age | 37.5 ± 10.9 | 38.3 ± 12.5 | 37.7 ± 12.7 | 0.809 |  | 35.8 ± 11.7 | 36.9 ± 11.8 | 36.5 ± 11.4 | 0.873 |  | 0.556 |
| Female sex | 27 (81.8) | 224 (77.5) | 576 (76.3) | 0.720 |  | 24 (72.7) | 89 (80.2) | 411 (78.6) | 0.657 |  | 0.913 |
| Presence of aura | 4 (12.1) | 53 (18.3) | 123 (16.3) | 0.564 |  | 5 (15.2) | 30 (27.0) | 98 (18.7) | 0.109 |  | 0.188 |
| Chronic migraine | 14 (42.4) | 74 (25.6) | 244 (32.3) | 0.038 |  | 10 (30.3) | 34 (30.6) | 162 (31.0) | 0.995 |  | 0.121 |
| Unilaterality | 23 (69.7) | 213 (73.7) | 580 (76.8) | 0.409 |  | 29 (87.9) | 95 (85.6) | 423 (80.9) | 0.335 |  | 0.815 |
| Pulsatility | 22 (66.7) | 221 (77.0) | 548 (73.9) | 0.337 |  | 24 (72.7) | 87 (78.4) | 404 (77.2) | 0.793 |  | 0.366 |
| Aggravation by or avoidance of physical activity | 30 (90.9) | 268 (93.1) | 647 (87.8) | 0.047 |  | 29 (87.9) | 97 (87.4) | 457 (87.4) | 0.996 |  | 0.105 |
| Nausea | 30 (90.9) | 259 (89.9) | 650 (87.4) | 0.460 |  | 30 (90.9) | 99 (89.2) | 472 (90.2) | 0.932 |  | 0.706 |
| Vomiting | 16 (48.5) | 138 (47.9) | 328 (44.1) | 0.503 |  | 20 (60.6) | 48 (43.2) | 275 (52.6) | 0.113 |  | 0.489 |
| Photophobia | 15 (45.5) | 165 (57.3) | 350 (47.1) | 0.012 |  | 12 (36.4) | 53 (47.7) | 261 (49.9) | 0.310 |  | 0.031 |
| Phonophobia | 26 (78.8) | 226 (78.5) | 549 (73.8) | 0.263 |  | 27 (81.8) | 81 (73.0) | 374 (71.5) | 0.432 |  | 0.133 |

Data were presented as n (%) or mean ± SD. T/C: TC or CT heterozygous group; C/C: CC homozygous group; significant level set at 0.05/11=0.005 after Bonferroni corrections for multiple comparisons.

**Table 2d. Migraine endophenotypes and *UPP2* rs7565931 genotyping**

|  | **C/C**  n=51 | **C/G or G/C**  n=360 | **G/G**  n=666 | *p value* |
| --- | --- | --- | --- | --- |
| Age | 37.7 ± 12.9 | 37.3 ± 12.7 | 38.2 ± 12.5 | 0.560 |
| Female sex | 38 (74.5) | 266 (73.9) | 523 (78.5) | 0.226 |
| Presence of aura | 8 (15.7) | 60 (16.7) | 112 (16.8) | 0.978 |
| Chronic migraine | 19 (37.3) | 102 (28.3) | 211 (31.7) | 0.322 |
| Unilaterality | 38 (74.5) | 271 (75.3) | 507 (76.1) | 0.934 |
| Pulsatility | 40 (78.4) | 258 (72.9) | 493 (75.0) | 0.606 |
| Aggravation by or avoidance of physical activity | 43 (84.3) | 323 (91.8) | 579 (88.4) | 0.127 |
| Nausea | 46 (90.2) | 311 (87.6) | 582 (88.3) | 0.851 |
| Vomiting | 21 (41.2) | 176 (49.6) | 285 (43.2) | 0.129 |
| Photophobia | 25 (49.0) | 181 (51.0) | 324 (49.2) | 0.863 |
| Phonophobia | 42 (82.4) | 270 (76.1) | 489 (74.2) | 0.389 |

Data were presented as n (%) or mean ± SD. C/C: CC homozygous group; G/C: GC or CG heterozygous group; G/G: GG homozygous group; significant level set at 0.05/11=0.005 after Bonferroni corrections for multiple comparisons.

**Table 2e. Migraine endophenotypes and *GPR39* rs10803531 genotyping**

|  | **C/A or C/A**  n=79 | **CC**  n=998 | *p value* |
| --- | --- | --- | --- |
| Age | 39.9 ± 11.6 | 37.7 ± 12.7 | 0.134 |
| Female sex | 56 (70.9) | 771 (77.3) | 0.197 |
| Presence of aura | 13 (16.5) | 167 (16.7) | 0.949 |
| Chronic migraine | 31 (39.2) | 301 (30.2) | 0.092 |
| Unilaterality | 55 (69.6) | 761 (76.3) | 0.185 |
| Pulsatility | 53 (68.8) | 738 (74.9) | 0.238 |
| Aggravation by or avoidance of physical activity | 74 (96.1) | 871 (88.8) | 0.045 |
| Nausea | 66 (85.7) | 873 (88.4) | 0.489 |
| Vomiting | 30 (39.0) | 452 (45.7) | 0.249 |
| Photophobia | 38 (49.4) | 492 (49.8) | 0.933 |
| Phonophobia | 62 (80.5) | 739 (74.8) | 0.263 |

Data were presented as n (%) or mean ± SD. C/A or A/C: CA or AC heterozygous group; C/C: CC homozygous group; significant level set at 0.05/11=0.005 after Bonferroni corrections for multiple comparisons.

**Table 3. Allodynia symptoms during migraine attacks**

|  |  | Total |  | *TRPM8 rs10166942* | | | | |
| --- | --- | --- | --- | --- | --- | --- | --- | --- |
|  |  |  |  | T/T or T/C |  | C/C |  | *p* value |
| Combing your hair |  | 131 (15.8) |  | 87 (17.6) |  | 44 (13.3) |  | 0.095 |
| Pulling your hair back |  | 271 (32.8) |  | 171 (34.5) |  | 100 (30.1) |  | 0.184 |
| Shaving your face |  | 20 (2.4) |  | 16 (3.2) |  | 4 (1.2) |  | 0.063 |
| Wearing eyeglasses |  | 223 (27.0) |  | 148 (29.9) |  | 75 (22.6) |  | **0.020^*^** |
| Wearing contact lenses |  | 183 (22.0) |  | 125 (25.3) |  | 58 (17.5) |  | **0.008^**^** |
| Wearing earrings |  | 80 (9.7) |  | 60 (12.1) |  | 20 (6.0) |  | **0.004^*^** |
| Wearing necklaces |  | 80 (9.7) |  | 62 (12.5) |  | 18 (5.4) |  | **0.001^**^** |
| Wearing anything on head or neck |  | 222 (26.8) |  | 140 (28.3) |  | 82 (24.7) |  | 0.254 |
| Wearing anything on forearm or wrist |  | 115 (13.9) |  | 86 (17.4) |  | 29 (8.7) |  | **<0.001^**^** |
| Wearing a ring |  | 75 (9.1) |  | 56 (11.3) |  | 19 (5.7) |  | **0.006^**^** |
| Wearing watch |  | 106 (12.8) |  | 70 (14.1) |  | 36 (10.8) |  | 0.164 |
| Wearing tight clothes |  | 306 (37.0) |  | 192 (38.8) |  | 114 (34.3) |  | 0.194 |
| Put on heavy quilt |  | 134 (16.2) |  | 88 (17.8) |  | 46 (13.9) |  | 0.133 |
| When shower water hits your face |  | 110 (13.3) |  | 77 (15.6) |  | 33 (9.9) |  | **0.020^*^** |
| Resting your face on the pillow on the side of the headache |  | 221 (26.7) |  | 132 (26.7) |  | 89 (26.8) |  | 0.964 |
| Exposure to heat (e.g. cooking) |  | 203 (24.5) |  | 140 (28.3) |  | 63 (19.0) |  | **0.002^**^** |
| Breathing through your nose on a cold day |  | 129 (15.6) |  | 88 (17.8) |  | 41 (12.3) |  | **0.035^*^** |

Data were presented as n (%) or mean ± SD as indicated. ^*^*p* < 0.05; ^**^*p* < 0.01; because of high collinearity for items listed in the allodynia questionnaire, we did not perform corrections for multiple comparisons to avoid type II error.
